# Endogenous retroviruses make aging go viral

**DOI:** 10.1093/lifemedi/lnad001

**Published:** 2023-01-10

**Authors:** Sha Zhou, Lin Liu, Xinyi Lu

**Affiliations:** State Key Laboratory of Medicinal Chemical Biology, Nankai University, Tianjin 300071, China; State Key Laboratory of Medicinal Chemical Biology, Nankai University, Tianjin 300071, China; College of Life Science, Nankai University, Tianjin 300071, China; State Key Laboratory of Medicinal Chemical Biology, Nankai University, Tianjin 300071, China

Endogenous retroviruses (ERVs) originate from integrated exogenous retroviruses and become part of the host genome during evolution. It now seems that they are hallmarks of aging and responsible for the spreading of cellular senescence in human.

**Figure 1.**
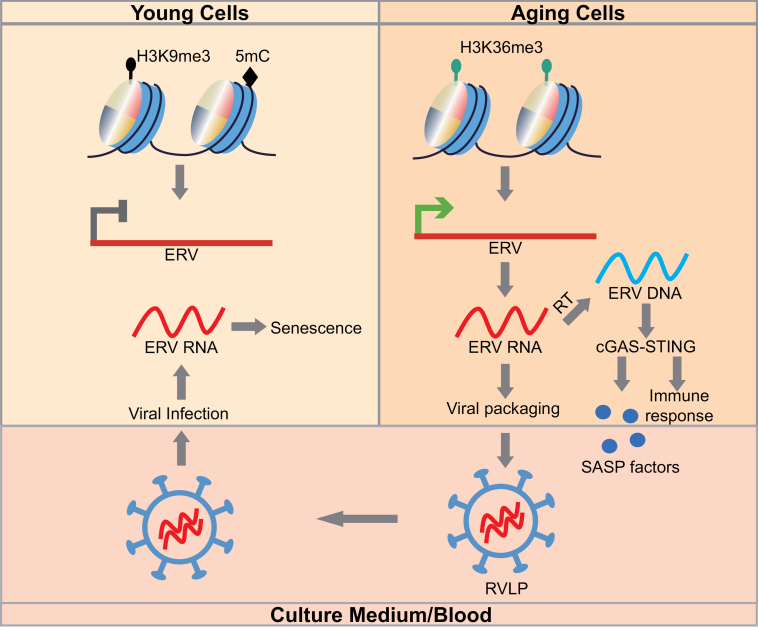
ERV RLVPs produced by senescent cells promote senescence and induce the aging of young cells. In young cells, ERVs are repressed by H3K9me3 and DNA methylation. In aging cells, ERV activation is associated with an increment of H3K36me3 and the absence of H3K9me3 and DNA methylation. Upon ERV transcription, ERV RNA can either be packaged with viral proteins into RVLPs or be reverse transcribed (RT) into ERV DNA. The cGAS-STING pathway recognizes ERV DNA and induces innate immune responses and SASP factors. RVLPs in culture medium or blood can infect young cells and induce cellular senescence.

Aged tissues often contain senescent cells, which no longer have the ability to properly function and proliferate. If senescent cells resist clearance and linger in tissues, they produce senescence-associated secretory phenotype (SASP) factors that spread inflammation and damage to other cells. However, other drivers of the spread of senescence remain obscure. In a recent issue of *Cell*, Liu et al. [1] discover ERV as a key driver of cellular senescence and tissue aging (Fig. 1).

A significant fraction of the human genome is composed of sequences derived from ERVs, which are remnants of ancient exogenous retroviral infections. Many ERVs have lost the ability to produce infectious viruses during evolution, but some evolutionarily young ERVs, such as HERVK, preserve the genes required for viral particle production [2]. ERVs are tightly controlled by healthy host cells to maintain genome stability, but they can be reactivated in senescent cells [3]. However, the function of these activated ERVs during aging is still unexplored.

Liu et al. cleverly designed experiments by taking advantage of the Huchinson-Gildford progeria syndrome (HGPS) and Werner syndrome (WS) models to study the expression and function of ERVs during aging [1]. They found that HERVK transcripts, viral proteins, and HERVK retrovirus-like particles (RVLPs) were highly activated in prematurely aged human mesenchymal progenitor cells (hMPCs) derived from HGPS and WS patients. This was similarly observed in aged human primary fibroblasts and hMPCs. They also discovered that decreasing silencing epigenetic marks DNA methylation and H3K9me3 while increasing H3K36me3 likely enabled HERV-K expression. To study the function of activated HERVK, the group adopted the CRISPR activation (CRISPRa) system to trigger HERVK transcription while using CRISPR inhibition (CRISPRi) and shRNAs to suppress HERVK expression. Notably, HERVK activation led to senescence in hMPCs, whereas suppression of HERVK hindered premature aging in HGPS and WS hMPCs. HERVK is similarly activated upon the inhibition of DNA methylation with 5-azacytidine (5-aza), whilst the cellular senescence induced by 5-aza can be partially rescued by HERVK depletion. These findings demonstrate a critical role for HERVK in the regulation of senescence.

Senescent cells often accumulate cytoplasmic DNA, which can be sensed by the cGAS-STING pathway [4]. The cGAS-STING signaling axis is a virus-fighting mechanism that activates innate immunity and SASP. To investigate how HERVK expression causes cellular senescence, the authors checked the immune responses after HERVK activation [1]. They found HERVK DNA accumulation in the cytoplasm of senescent hMPCs. This is accompanied by the activation of cGAS-STING-mediated innate immune response. Both the inhibition of HERVK reverse transcription and the depletion of HERVK or STING alleviated cellular senescence by attenuating the immune response and SASPs. In contrast, activation of HERVK by CRISPRa or 5-aza triggered immune responses and the production of SASP cytokines in young hMPCs. As a result, the authors identified cGAS-STING downstream of HERVK to regulate cellular senescence.

Next, Liu et al. elegantly showed that HERVK RVLPs, which were released into culture medium by senescent cells, were capable of infecting young hMPCs and accelerating senescence in these cells by activating cGAS-STING signaling [1]. In addition, infecting young hMPCs with packaged HERVK RVLPs resulted in the same senescent phenotype. The blockage of senescence induction by immunodepletion of HERVK RVLPs validated the direct role of HERVK RVLPs in spreading senescence to other cells. The authors further tested the possibility of using ERVs as aging biomarkers in humans and monkeys. They found that ERVK and ERVW, as well as the innate immune response, were activated in various tissues of aged cynomolgus monkeys and the HGPS monkey model. They also found that HERVK in the serum of aged humans induced aging phenotypes in primary hMPCs, suggesting that ERVs can be used as aging biomarkers.

In their final set of experiments, Liu et al. used aged mice to assess the possibility of targeting ERVs in the reversal of tissue aging [1]. They found that mouse mammary tumor virus (MMTV) and immune responses were activated in aged mice. Suppression of MMTV expression with CRISPRi or a reverse transcriptase (RT) inhibitor (Abacavir) rescued cartilage degeneration and grip strength. Remarkably, Abacavir treatment can even improve overall physical condition and short-term memory in aged mice, implying ERVs as potential therapeutic targets against organismal aging.

The work by Ref. [1] addresses several important points with regard to the role of ERVs in the aging process. First, these intriguing findings establish a direct link between ERVs and senescence. This expands our current understanding of molecular regulators of aging. In addition, these findings establish a clear mechanistic link between ERVs and the spreading of cellular senescence, proposing a novel way for senescent cells to influence healthy tissues. Furthermore, this discovery is incredibly important in light of identifying ERVs as potential therapeutic targets in organismal aging. By providing new strategies and development bases for delaying aging, this work contributes significantly to the research and development of the field of aging.

The observations by Ref. [1] also raise several intriguing questions. One route to pharmacologically disrupt ERVs is using the RT inhibitor Abacavir, which has been used for the treatment of HIV infection. As a senomorhpic compound, the application of Abacavir against senescence would require continuous administration. However, cellular senescence is also a tumor-suppressive mechanism to prevent the propagation of mutations in damaged or transformed cells within aged tissues. Therefore, it is necessary to assess the long-term impact of Abacavir treatment before moving into translational application among the aged population. It is interesting to observe a positive feedback regulation of cellular senescence by HERVK. However, HERVK is occasionally activated and eventually suppressed under physiological conditions, e.g. in human embryonic cells [5]. Thus, it would be fascinating to probe the possibility of mimicking physiological conditions in order to turn off the positive feedback between HERVK and senescence. Another exciting observation is that ERVs are hallmarks of aging in different species, including human, primate, and mouse. However, the level of ERV RLVPs in blood needs to be qualified for using ERVs as aging clocks. Hence, future quantification of the absolute physiological level of ERVs across a broad population of various ages might provide further insights into the relationship between ERVs and organismal age.
